# Crowd counting at the edge using weighted knowledge distillation

**DOI:** 10.1038/s41598-025-90750-5

**Published:** 2025-04-08

**Authors:** Muhammad Asif Khan, Hamid Menouar, Ridha Hamila, Adnan Abu-Dayya

**Affiliations:** 1 Qatar Mobility Innovations Center, Qatar University, Doha, Qatar; 2 Electrical Engineering, Qatar University, Doha, Qatar

**Keywords:** Computational science, Computer science

## Abstract

Visual crowd counting has gained serious attention during the last couple of years. The consistent contributions to this topic have now solved several inherited challenges such as scale variations, occlusions, and cross-scene applications. However, these works attempt to improve accuracy and often ignore model size and computational complexity. Several practical applications employ resource-limited stand-alone devices like drones to run crowd models and require real-time inference. Though there have been some good efforts to develop lightweight shallow crowd models offering fast inference time, the relevant literature dedicated to lightweight crowd counting is limited. One possible reason is that lightweight deep-learning models suffer from accuracy degradation in complex scenes due to limited generalization capabilities. This paper addresses this important problem by proposing knowledge distillation to improve the learning capability of lightweight crowd models. Knowledge distillation enables lightweight models to emulate deeper models by distilling the knowledge learned by the deeper model during the training process. The paper presents a detailed experimental analysis with three lightweight crowd models over six benchmark datasets. The results report a clear significance of the proposed method supported by several ablation studies.

## Introduction

Unmanned Aerial Vehicles (UAVs) using visual computing enable automated crowd surveillance and analysis^1,2^ during mega-events, transportation planning, and search and rescue missions. An interesting application is to automatically collect and analyze crowd statistics (e.g., crowd-counting and density estimation) in a geographical area. Deep learning serves a major role in the automated analysis of crowd images or videos. However, the deployment of deep learning models on edge devices such as drones poses unique challenges due to limited computational resources, power constraints, and slow inference^3^. Though several lightweight crowd-counting models have been proposed during the last few years for resource-constraint devices like drones^2,4^, the accuracy of such models significantly degrades in complex scenes due to limited learning capabilities. Recent studies have shown that lightweight models suffer from large generalization errors with poor adaptation capabilities. Thus, when a model trained on a given scene/dataset is deployed in a new scene/dataset, the accuracy drops.

**Fig. 1:**
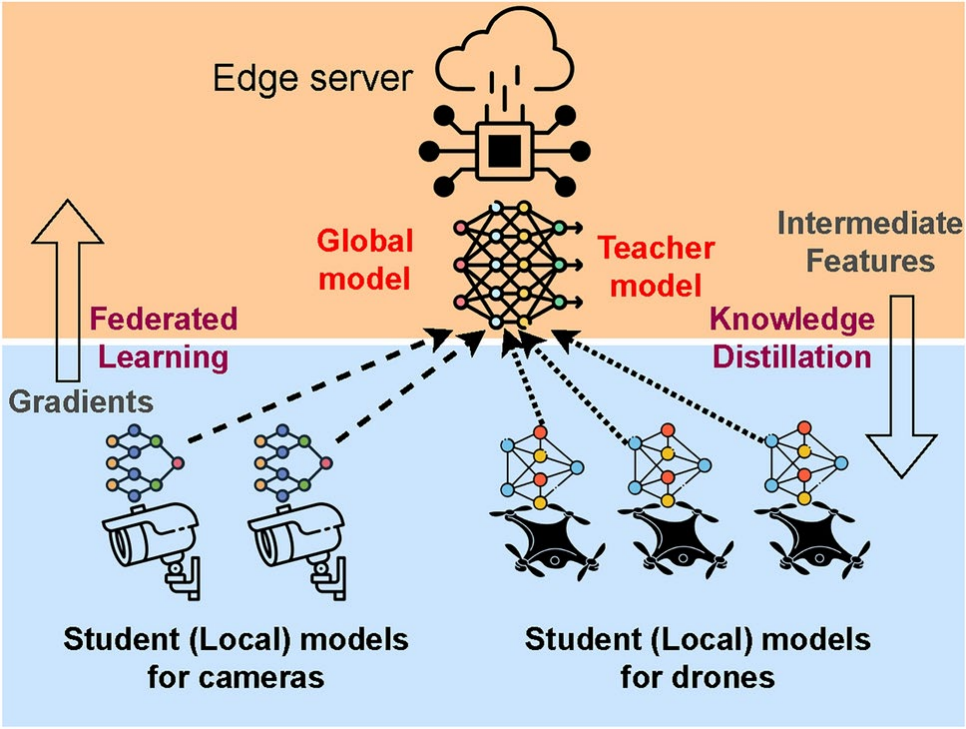
Collaborative training of multiple local models (student models) running over edge devices and a single global model (teacher model) running over an edge server via knowledge distillation.

To address these challenges, this paper explores the application of knowledge distillation (KD) to train lightweight models tailored for deployment on drones. Knowledge distillation^5^ is a model compression method that allows the transfer of learned knowledge from a cumbersome (deep) model to a lightweight (shallow) model. In the context of knowledge distillation, the cumbersome (deep) model is called the “teacher” model, whereas the lightweight ( shallow) model is referred to as the “student” model. It is argued that by distilling the knowledge learned by the teacher into the student, it is possible to achieve a significant reduction in model size and computational complexity without compromising performance^5^. The integration of knowledge distillation into the training process for lightweight models is a promising avenue to overcome resource limitations while maintaining or even enhancing the model’s effectiveness. Figure 1 depicts the potential scenario of training compact models using KD. The figure also highlights how KD framework can be integrated with federated learning (FL) scenarios in which the model updates flow in the opposite direction.

Over the last few years, KD has gained significant attention in the computer vision community which resulted in strong theoretical foundations and experimental studies in several vision problems. However, the applications of KD in crowd counting and density estimation have been untouched. Thus, it is interesting as well as useful to unveil the potential of KD in this significant application. This paper delves into the intricacies of knowledge distillation techniques, illustrating how they can be adapted and optimized for compact crowd-counting models suitable for drone applications. This paper presents a detailed and thorough experimental study encompassing three (3) lightweight crowd-counting models trained over six (6) benchmark datasets using knowledge distillation. The study covers diverse data-centric scenarios and several ablation studies to confidently conclude the potential benefits that can be achieved and the limitations of KD in the given context.

The contribution of this paper is as follows:

- We propose knowledge distillation (KD) to train lightweight models for crowd density estimation. The KD loss is formulated which allows distillation at any intermediate layers across both teacher and student models.
- The proposed method is extensively evaluated using three baseline lightweight models and two cumbersome teacher models over six benchmark crowd datasets to evaluate performance gains using the proposed scheme.
- Ablation studies are conducted to validate the results in diverse scenarios e.g., cross-domain adaptation, and different-teacher models.

The rest of the paper is organized as follows: Section “Knowledge distillation” presents a detailed theoretical overview of the knowledge distillation, whereas section “Crowd counting” presents a brief background of crowd counting methods and developments. Section “Related work” discusses recent relevant studies on KD and crowd counting to identify the research gap that motivates this work. Section “Proposed scheme” presents the proposed KD framework for crowd density estimation, which is investigated using a thorough set of experimental analyses presented in section “Experiments”. The results are reported and discussed in section “Results and discussion”. Further validation of the results is provided using ablation study in section “Ablation study”. Lastly, conclusions are drawn in section “Conclusion”.

## Knowledge distillation

Knowledge Distillation (KD) refers to the mechanism in which the knowledge learned by a cumbersome and sophisticated model (teacher) is transferred to a shallow model (student) to improve the student model’s generalization performance. Using KD, the student can capture the finer details learned by the teacher instead of learning from the labels. The student model is the model that will be deployed for the application.

In its simpler form, the student model can mimic the performance of the teacher model by using the class prediction probabilities of the teacher called “soft targets”. The soft targets are calculated using the “temperature” parameter in the softmax function.

1 $$\begin{aligned} q_i = \frac{exp \left( \frac{z_i}{T} \right) }{\sum _{j} {exp \frac{z_j}{T}}} \end{aligned}$$

where *T* is the “temperature” parameter used to soften the probabilities.

**Fig. 2:**
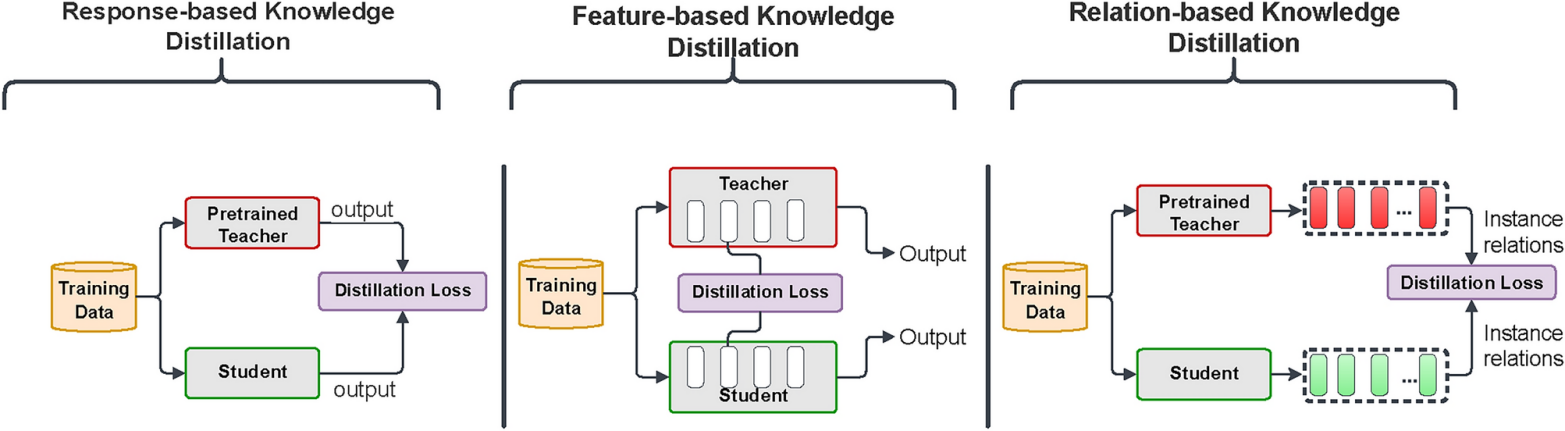
Three types of knowledge distillation (KD).

Knowledge can be distilled in three different ways. First, response-based KD^5^ in which the student model aims to mimic the teacher’s predictions, with the help of a distillation loss function that measures the difference between their logits. As the loss decreases over time, the student becomes more accurate in making predictions similar to the teacher. Second, feature-based distillation^6^ in which the teacher model, distills the data knowledge in its intermediate layers into the student models. The student model thus learns the same feature activations as the teacher. This is achieved by minimizing the difference between the feature activations of both models. Third, relation-based distillation^7^ in which connections between different layers or data samples are used employing a Flow of Solution Process (FSP) based on Gram matrices between layers to explore relationships among feature maps. The FSP matrix summarizes these relationships through inner product calculations between feature maps. Singular value decomposition is used to distill knowledge, with correlations between feature maps representing the distilled knowledge. This approach encapsulates relationships through feature maps, graphs, similarity matrices, feature embeddings, and probabilistic distributions, enhancing our understanding of knowledge distillation. Figure 2 illustrates the three types of KD in neural networks^8^. investigates KD and observed that a student model trained via KD can exhibit higher intra-class variance than the teacher model, and thus act as a better teacher^9^. have proposed an evolutionary knowledge distillation mechanism in which the pretrained teacher network is replaced by an online learned teacher model^9^. also consider training multiple teacher networks online synchronously, which all distill knowledge to the student and improves its performance^10^. propose a unique type of KD that uses heuristics to let the student learn from the teacher’s failures. This can also be applied to self-distillation.

## Crowd counting

Traditionally, crowd counting aims to detect simply the total object (e.g., people) count in an image^11^. These methods manually apply a feature detector to extract features such as full body shape^12^, or body parts^13–16^ and then apply ML models such as linear or ridge regression, support vector machines (SVMs), random forest (RF), gradient methods, or multi-layer perceptrons (MLPs) to predict the total count. These methods underperform in dense crowds due to occlusions, low resolution, and perspective distortions. Traditional methods regress the total count in an image^17^ or image patch^18^ using global features such as texture^19^, foreground^20^, gradients^21^. However, these methods provide poor estimates of high-density crowd images. Authors in^22^ used an alternative approach to divide images into patches, classify patches as crowd/no-crowd using SVM, and then count heads using head detection CNN network. The authors further proposed an enhancement in^23^ of the method in^22^, by classifying patches as low, medium, high-dense, or no-crowd. The patches of different density levels are then zoomed accordingly before regressing the count in them.

The most recent methods for crowd counting use density map estimation in which the input is an image and the output is the density map of the crowd^24,25^. The pixel values in the density map are summed up to get the total head count. Thus, a density map contains additional location information along with the total head count of the crowd scene. Density estimation typically uses convolution neural networks (CNNs)^26–34^. CNNs offer a strong capability of automatic feature extraction and have shown remarkable performance in several computer vision problems such as image classification^35,36^, object detection^37^, and image segmentation^38^. CNNs can be used for both global count estimation as well as density map estimation^39^. A two-stage crowd counting has been proposed in^29^ to regress both local density and global count. Similarly^40^, proposed a cascaded architecture of a density map regressor and a global crowd count regressor (using weak supervision). The global count regressor learns scene-level features, whereas the density map regressor effectively learns features such as shape variation and background effects. The multistream convolutional neural network (AMS-CNN) is proposed in^41^ to learn features using three streams and then fuses the spatial, temporal, and spatial foreground features from different cues of the crowd scene^42^. proposes a multi-attention Spatial-Temporal CNN architecture to learn cluttered background and scale variation in crowd scenes. Scale variation and background noise and clutters are challenging problems in crowd counting and several works have been published recently to address the issue. For instance^43^, proposed a novel architecture ($SA^2Net$) to address these challenges by proposing a multiscale feature aggregator (MFA) module and a background noise suppressor (BNS). The $SA^2Net$ uses a composite loss (global consistency loss and Euclidean loss) to optimize the network. Similarly^44^, further proposed FPANet to solve the scale variation problem in lightweight models through special attention modules by suppressing misleading information. Authors in^45^ addressed the scale variation challenge using a different approach by jointly solving the counting and localization problems, using a combined Euclidean and structural similarity loss function. Emerging concepts such as quantum machine learning have been used in^34^ for crowd counting for dense crowd counting with extreme background noise to boost the model’s generalization capabilities. Recently^46^, proposed incremental learning for crowd object counting for improved learning of new object classes. The images are annotated by humans by placing a dot on the head position, there might be small errors in the annotation, however such errors (if not significantly high) do not significantly impact the accuracy much^47^. When using knowledge distillation, the teacher model, which has a higher capacity and is trained on a larger set of labeled data, helps reduce the impact of annotation noise when transferring knowledge to the student model. By using soft labels and intermediate feature maps from the teacher model, the student model gains a more robust representation, which can help it generalize better even when training data includes some level of human error. The performance of the shallow crowd models is significantly impacted by scale variations in head sizes, background clutters, illumination variations, and occlusions. However, the method distill knowledge from a deeper model trained with multi-scale features to help the shallow model to cope with scale variations, and other challenges such as background clutters and occlusions. The teacher model captures and transfers these scale-aware features to the student model, enabling it to better handle variations in object size within the scene.

## Related work

Knowledge distillation (KD) in neural networks was proposed by Hinton et al. in^5^ which is inspired by the work^48^ demonstrating that “knowledge learned by a large ensemble of models can be transferred to a single model”. Hinton et al.^5^ introduced the “temperature” parameter *T* in the softmax function at the output layer used to distill the knowledge from the teacher model to the student model by training the student model using the same dataset as used for training the teacher model with a high value of *T*. Once the student model is trained, the value of *T* is set to $T=1$ (standard softmax function) during the inference. The student model’s accuracy can be highly improved by using two objective (loss) functions i.e., a cross-entropy (CE) function with the soft targets (produced by the teacher) and a CE with the actual ground truth labels. The authors suggest using a high value of *T* in the first case (same *T* in student and teacher) and $T=1$ for the second case. However, the method was limited to supervised learning. To extend the KD concept to any type of deep learning model, Romero et al.^6^ proposed feature-based KD to learn the intermediate representation (activation maps). They proposed to match the intermediate layer outputs of the teacher and student models to guide the student model. Several works extended this model to propose different methods to match feature activation maps such as using attention maps^49^, neuron selectivity^50^, and factors^51^. Similar to the response-based KD in which the knowledge is distilled from the output layer, in feature-based KD the knowledge is distilled from a single intermediate layer. In contrast,^7^ proposed relations-based KD in which a Gram matrix is calculated as an inner product between the feature maps of two layers. The model has been further extended by^52^ to learn from more than one teacher.

Authors in^53^ highlighted that the transfer of knowledge is impacted by the difference in the capacity of the teacher and student models. Knowledge distillation from multiple teacher networks is proposed in^54^. In this work, the authors used two teacher networks, where one teacher network distills knowledge from intermediate feature maps to the student (feature-based KD), whereas the second teacher network directly distills the output probabilities (response-based KD). Knowledge distillation can also be used alongside other model compression techniques. For example, authors in^55^ propose knowledge distillation with quantization. Similarly, authors in^56^ suggest incorporating pruning with knowledge distillation.

The aforementioned works illustrate the benefits and different approaches to KD in neural networks mostly in classification tasks. KD typically performs well in classification due to the so-called “dark knowledge” in the softened logits of the teacher which the student uses to emulate the generalization capability of the teacher^57^. On the contrary, KD in image regression tasks that involve predicting continuous and unbounded outputs can be limited^58^. KD has been used in a few regression tasks such as pose estimation^57^, and gaze estimation^59^. Recently, KD has been applied in crowd counting based on the density estimation method. For instance, authors in^60^ applied KD in crowd counting to train a shallow network (EdgeCount) using a teacher network (EdgeCount_T). Both EdgeCount and EdgeCount_T use the same architecture i.e., encoder-decoder architecture using MobileViT^61^ as the encoder. EdgeCount^60^ uses extra modules such as spatial channel reconstruction convolution (SCConv) composed of spatial reconstruction unit (SRU) and channel reconstruction unit (CRU) and a low parameter weighted multi-scale feature fusion module (LWMFFM) to enhance the model performance. Similarly^62^, proposes using self-distillation for crowd counting to help a crowd model using previous meaningful knowledge. Self-distillation can help the model to retain the most recent knowledge, but it can not be used to make shallow model learns more features from deeper models. Authors in^63^ used KD to distill foreground knowledge from a density map to enhance counting performance in challenging scenarios. This method does not use KD to train shallow networks.

This paper studies and evaluates through extensive experiments the benefits of KD in model-agnostic (different student/teacher) settings using multiple teacher and student models, on cross-scene datasets of different crowd data (humans, cars, animals).

## Proposed scheme

**Fig. 3:**
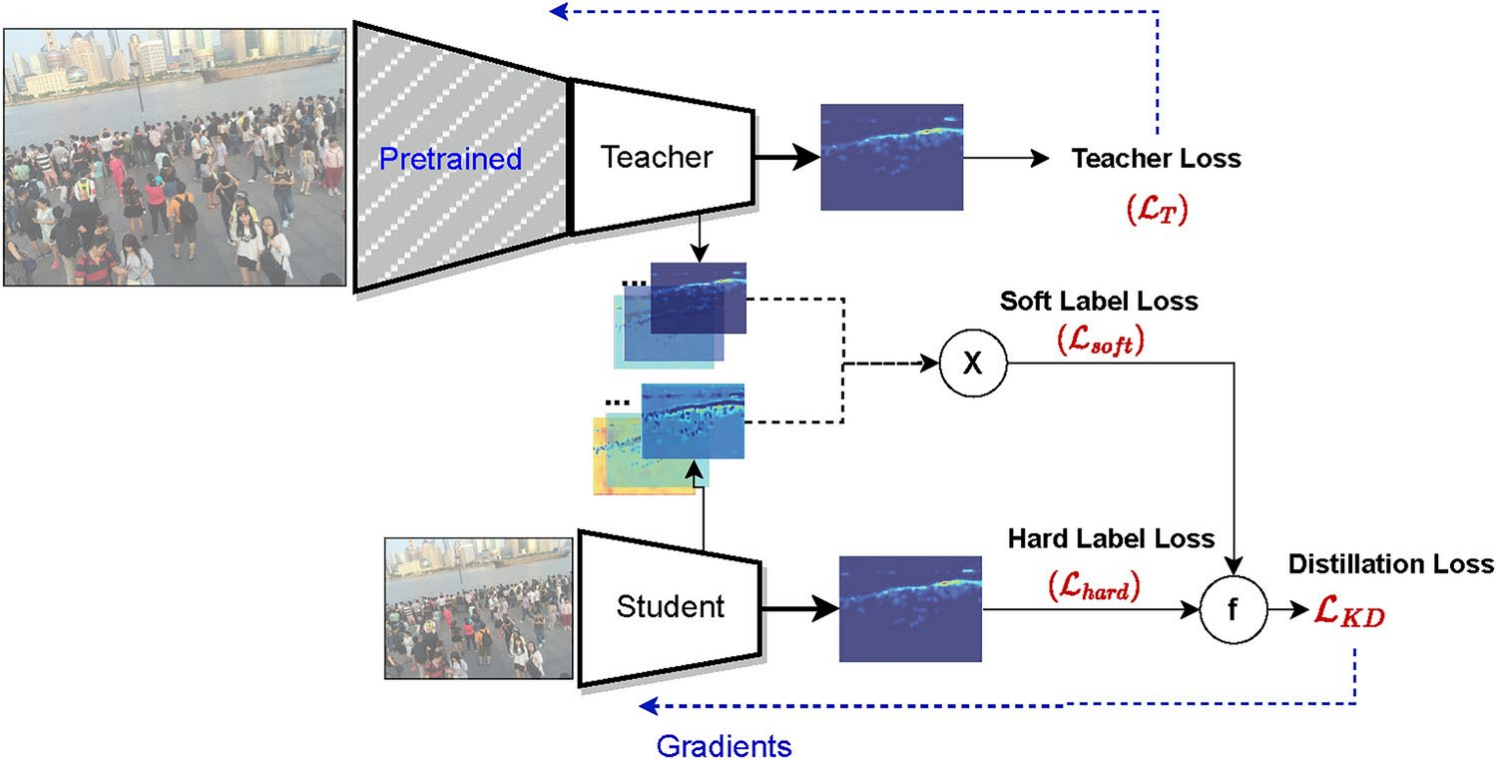
Proposed scheme using knowledge distillation from a deeper teacher network into a smaller student network.

The proposed method (Fig. 3) is based on knowledge distillation (KD). KD originally has been defined as a logits-matching problem for classification. Let *T* be the teacher model, and *S* be the student model, both generating outputs $O_T = softmax(a_T)$, and $O_S = softmax(a_S)$, respectively. $a_T$ and $a_S$ are the pre-softmax logits and $O_T$ and $O_S$ are the class probabilities of *T* and *S* models, respectively.

Since the teacher outputs are usually very close to true labels (assuming *T* has a very high accuracy), a temperature variable $\tau$ is used to soften the outputs of *T*, and the same $\tau$ is used in the student model *S* as well during training. Then in the inference stage, *S* uses $\tau = 1$. The softened outputs potentially provide more information to the student model than the hard labels.

2 $$\begin{aligned} O_T^\tau = softmax \left( \frac{a_T}{\tau }\right) = \frac{ exp( \frac{a_i^T}{\tau }) }{ \sum _{i}exp(\frac{a_j^T}{\tau } ) } \end{aligned}$$

3 $$\begin{aligned} O_S^\tau = softmax \left( \frac{a_S}{\tau }\right) = \frac{ exp( \frac{a_i^S}{\tau }) }{ \sum _{i}exp(\frac{a_j^S}{\tau } ) } \end{aligned}$$

At high $\tau$ values, the distillation loss can be calculated as^5^:

4 $$\begin{aligned} {\mathscr {L}} = \frac{1}{2}(z_i - v_i)^2 \end{aligned}$$

where $z_i$ and $v_i$ are zero-meaned logits of the student and teacher models, respectively.

If the true labels are available, distillation loss is calculated as a weighted sum of two errors: (i) hard label error ${\mathscr {H}} (y,O_S)$ and (ii) soft label error ${\mathscr {H}}(O_T^\tau , O_S^\tau )$.

5 $$\begin{aligned} {\mathscr {L}}_{KD} = \alpha {\mathscr {H}} (y,O_S) + (1-\alpha ) {\mathscr {H}}(O_T^\tau , O_S^\tau ) \end{aligned}$$

where ${\mathscr {H}}$ is the cross entropy and *y* is the hard label or ground truth. The term $\alpha$ is a balancing parameter between the hard label error ${\mathscr {H}} (y,O_S)$ and the soft label error ${\mathscr {H}}(O_T^\tau , O_S^\tau )$. For instance, setting the value of $\alpha < 0.5$ means a higher weight is assigned to the soft label error.

In the density estimation problem, the model estimates the density map i.e., true pixel values rather than the sparse class labels. In the proposed system, the teacher model is trained on the labeled dataset to produce density maps. $T(x_i)$ for each input $x_i$. The student model is trained on the same labeled dataset but it also utilizes the predictions of the teacher model. The distillation loss function is typically based on the $L_2$ distance between the teacher’s predictions $T(x_i)$ and the student’s predictions $S(x_i)$. The student model is trained to minimize this loss function, aiming to match the teacher’s predictions. The temperature parameter is used to soften the predictions of both the teacher and the student models.

As the crowd scenes might be complex (e.g., dense crowds, clutters, complex background, etc.), the teacher model thus captures general trends under varying conditions and the student benefits from this knowledge, learning a more robust regression function that generalizes better across diverse scenarios. In such scenarios, the temperature parameter $\tau$ plays a crucial role. The temperature parameter $\tau$ in Eq. 5 directly influences the transfer of knowledge to the student model. Unlike classification tasks, in which the temperature smoothens the logits to emphasize the relative probabilities between classes, in regression, it smoothens the output distribution or modulates the gradients during training. This helps in capturing broader patterns rather than exact pointwise predictions. A higher temperature emphasizes learning the overall trends or correlations in the data as provided by the teacher, rather than overfitting the fine-grained teacher outputs, whereas, a lower temperature makes the student model mimic the teacher more closely, which could lead to overfitting to the teacher’s noise or inaccuracies. In other words, a higher value of the temperature parameter leads to better generalization, whereas a lower value enforces precision in mimicking the teacher’s outputs, which might be advantageous when the teacher model is highly accurate and represents the desired regression function well. In the proposed KD-based regression framework, the temperature parameter scales gradients, influencing how much the student prioritizes the teacher’s predictions over the ground truth data. Thus, the temperature parameter needs finetuning to help the student model converge more effectively by balancing the trade-off between learning from ground truth data and the teacher’s knowledge.

We formulate the loss function for density map distillation as follows^57^:

6 $$\begin{aligned} {\mathscr {L}}_{reg} = \frac{1}{n} \sum _{i=1}^{n}{ \alpha \left\| p_S - p_{GT} \right\| ^2 + (1-\alpha ) \phi _i \left\| p_S - p_T \right\| ^2} \end{aligned}$$

In eq. 6, $\phi$ is the normalized teacher loss and is calculated as follows:

7 $$\begin{aligned} \phi _i = \left( 1- \frac{\left\| p_T - p_{GT} \right\| ^2}{\eta } \right) \end{aligned}$$

where ${\mathscr {L}}_T$ is the $L_2$ loss of the teacher over the train dataset.

8 $$\begin{aligned} \eta= & max({\mathscr {L}}_T) - min({\mathscr {L}}_T) \end{aligned}$$

9 $$\begin{aligned} {\mathscr {L}}_T= & \left\| p_T - p_{GT} \right\| ^2 \quad \forall j \in N \end{aligned}$$

## Experiments

The proposed method is evaluated over 6 benchmark datasets and three well-known crowd models, explained in this section.

### Datasets

There are a number of publicly available crowd-counting datasets^64^ of various sizes and complexity. However, we carefully considered these and chose five datasets for specific reasons as explained in the following. We omitted the use of datasets with too congested scenes as the student models used in this study are too shallow and will certainly not achieve sufficient accuracy. It is worth noting that the aim of this study is to effectively apply KD to train extremely lightweight and shallow architectures ($\le$ 0.2 million parameters), which are suitable for deployment over resource-constrained edge devices in real-time applications and especially when the crowd estimate is required rather than the most accurate count.

#### Mall

The Mall dataset^65^ is a publicly available dataset containing 2000 video frames of fixed size (640x480 pixels) captured from a single webcam (single scene). The dataset contains a total of 60,000 pedestrians annotated. The dataset is chosen to evaluate the models’ performance in a single-scene CCTV deployment.

#### ShanghaiTech

The ShanghaiTech dataset^26^ is a widely used benchmark dataset tailored for crowd counting research. It features high-resolution images captured from diverse real-world scenes, primarily in the streets of Shanghai, China. The dataset comprises a total of 1198 images with 330,165 annotations. It is partitioned into two distinct parts: Part-A containing 482 images, and Part-B, containing 716 images. Within Part-A, there are train and test subsets, each containing 300 and 182 images, respectively. Similarly, Part-B is subdivided into train and test subsets, with 400 and 316 images, respectively. The dataset is chosen to evaluate the models’ performance in a cross-scene CCTV deployment.

#### CARPK

The Car Parking Lot (CARPK) dataset^66^ comprises images of car parking containing a total of 90,000 vehicles gathered from four distinct parking lots using a PHANTOM 3 PROFESSIONAL drone. The images were captured from an aerial perspective at an altitude of approximately 40 meters. Each image in the dataset is annotated with bounding boxes delineating the position of individual cars. The dataset facilitates tasks such as object counting, and object detection. The dataset is chosen to evaluate the models’ performance in a cross-scene drone deployment for vehicle counting.

#### DroneRGBT

The DroneRGBT dataset^67^ is a collection of images captured using a drone equipped with both RGB (Red, Green, Blue) and thermal (T) sensors. This dataset combines information from both visual and thermal spectrums, providing a rich source of data for various computer vision and remote sensing tasks. The images are obtained from aerial viewpoints, offering unique perspectives on different environments. The dataset is annotated and categorized to facilitate tasks such as object detection, classification, and semantic segmentation in both RGB and thermal domains. The dataset contains 3600 pairs of RGB and thermal images. The dataset is chosen to evaluate the models’ performance in a cross-scene drone deployment for people counting, especially in low light conditions.

#### Aerial sheeps

The aerial sheep dataset^68^ consists of 1727 images of sheep from a birds-eye view. The dataset is divided into train (1203 images), validation (350 images), and test (174 images) sets. All images are of fixed spatial resolution ($600 \times 600$). The dataset is chosen to evaluate the models’ performance in a cross-scene drone deployment for animal counting.

**Fig. 4:**
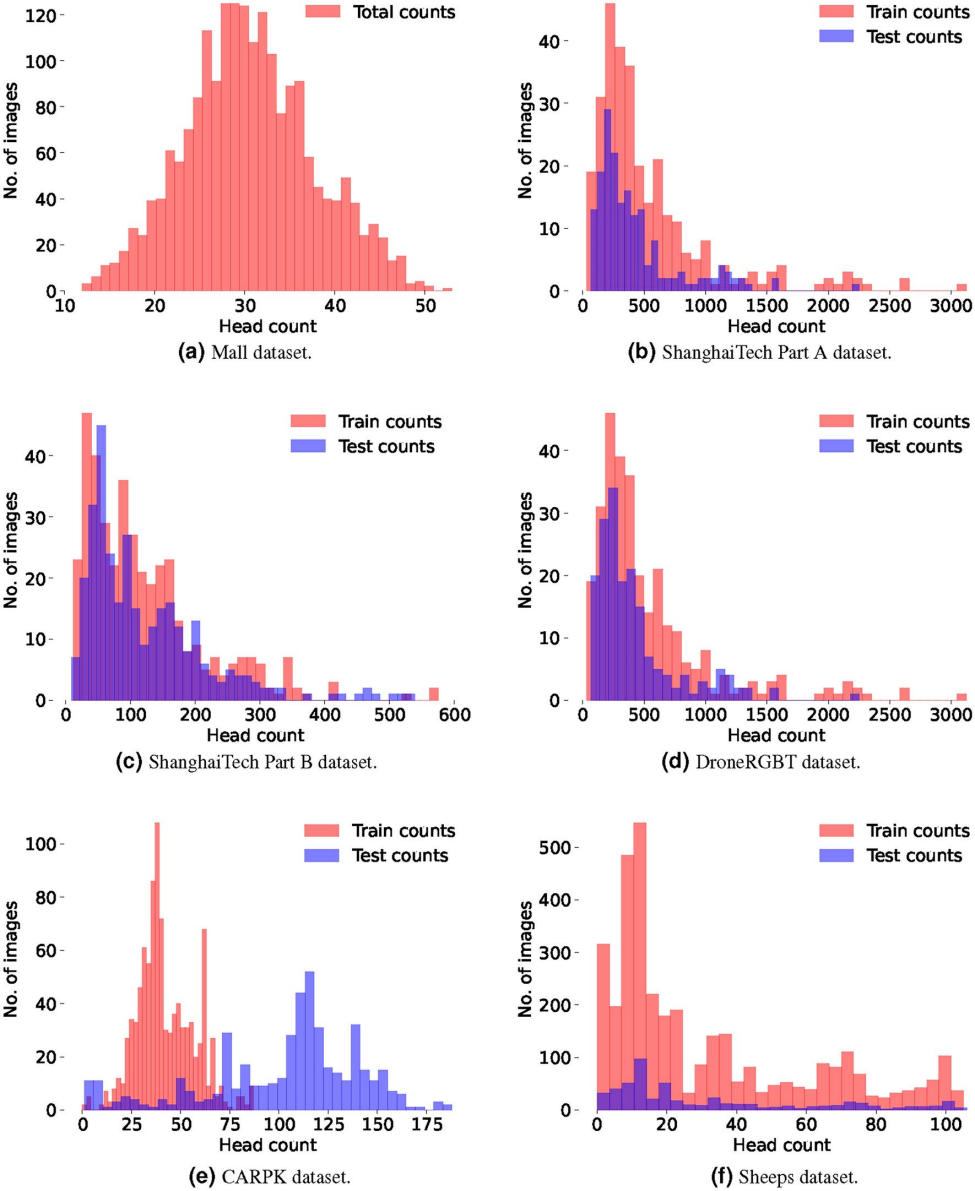
Count histograms of various datasets used in this study.

Figure 4 shows the count histograms of the six datasets used in this study.

### Baselines

We used two deep neural networks called CSRNet and CSRNet_lite^2^ that serve as teacher models, and three shallow networks i.e., MCNN, DroneNet^4^, LCDnet^2^ as student models.

#### MCNN

This multi-column CNN^26^ architecture uses adaptive kernels to capture perspective distortion in crowd images. The network consists of three columns of Conv layers with different sizes of filters. MCNN uses max pooling layers of $2 \times 2$ after each Conv layer and ReLU activation function. The outputs of the three columns are stacked to get the final density map. The spatial resolution of the final density map is one-fourth of the size of the input image.

#### DroneNet

The DroneNet crowd model^4^ uses the same three-column structure of MCNN with the Conv layers replaced with Self-ONN layers^69^. Self-ONN (Operational Neural Networks) layers perform non-linear operations to learn more fine-grained features.

#### LCDnet

The lightweight crowd density estimation (LCDnet) model^2^ is an extremely lightweight architecture proposed for aerial surveillance using drones. Since drones capture the top view of the scene, the perspective distortion arising from the camera field-of-view is minimal when the drone is at a constant altitude. LCDnet achieves comparable performance to MCNN^26^ on aerial images (e.g., DroneRGBT and CARPK datasets) with almost half the number of parameters. Also, the spatial resolution of the output density map in LCDnet is double of MCNN output.

The three models are chosen because these models are extremely lightweight with parameters less than 0.2 million (Table 2). All three models do not rely on transfer learning. The MCNN and DroneNet are both multicolumn architectures, but MCNN uses CNN layers with non-linear activation functions, whereas DroneNet uses non-linear self-ONN layers. The LCDnet uses a shallower hybrid architecture with only 0.05 million parameters.

**Fig. 5:**
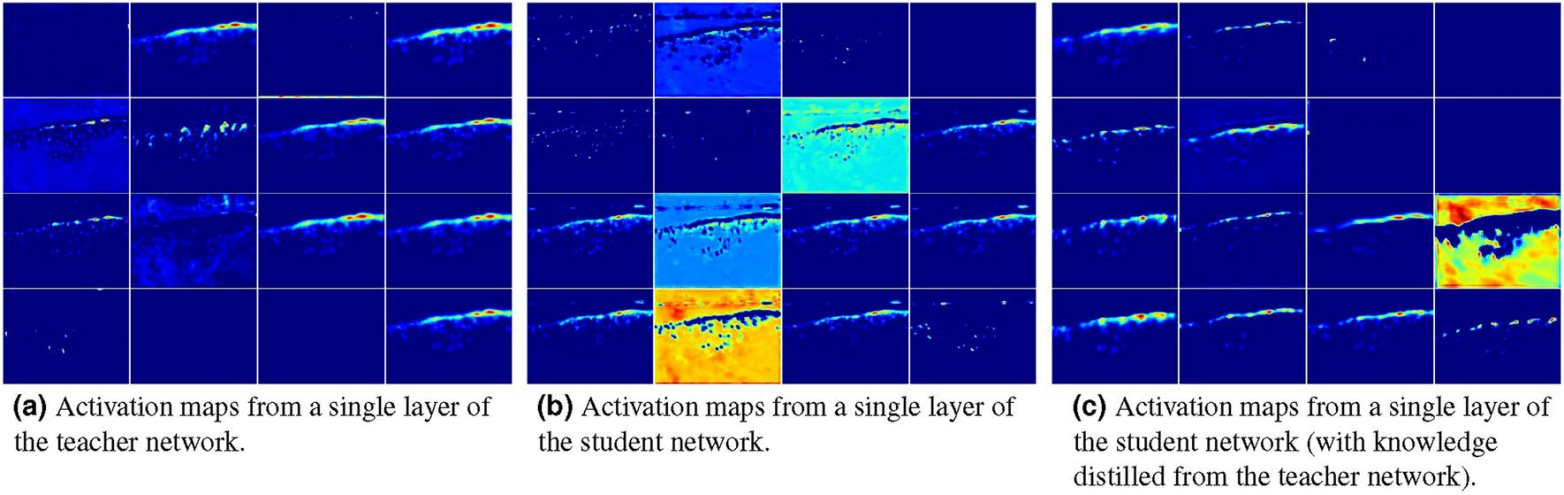
An illustration of activation maps obtained from the teacher and student network. This shows better learning ability of the teacher network which learned more fine-grained features. The student’s features are apparently less rich than the teacher network which are highly improved using the knowledge distilled from the teacher network.

### Training

We used the PyTorch framework to implement the proposed scheme. First, the annotations (localization maps) are converted into density maps using Gaussian kernel using the following Eq. 10.

10 $$\begin{aligned} D = \sum _{i=1}^{N}{ \delta (x-x_i) * G_\sigma } \end{aligned}$$

Eq. 10 convolves the dot annotations $\delta (x-x_i)$ with the Gaussian function $G_\sigma = \frac{1}{s \sqrt{2\pi }} e^{-\frac{(x - \mu )^2}{2\sigma ^2}}$ for all *N* (number of points i.e., head positions in the dot annotation matrix). For simplicity and reproducibility, we use fixed-size kernels (i.e., the scale parameter $\sigma$) for all the images in the same dataset. Specifically, the $\sigma =[10, 15, 7, 8, 10]$ are used for ShanghaiTech Part A, ShanghaiTech Part B, DroneRGBT, CARPK, and Mall datasets, respectively.

All models are trained on a Lambda machine with Quadro RTX-8000 GPUs. We use a constant learning rate of 0.001 in all experiments. the student models using KD use the loss function defined in Eq. 6, whereas the baseline models (students without KD) and the teacher model (without KD) use the standard training method using mean squared error (MSE) as a loss function as defined in Eq. 11.

11 $$\begin{aligned} {\mathscr {L}}(\Theta ) = \frac{1}{N} \sum _{1}^{N}{ ||D(X_i;\Theta ) - D_i^{gt}||_2^2} \end{aligned}$$

where *N* is the number of samples in the training set, $D(X_i;\Theta )$ is the model estimated density map for the input image $X_i$, and $D_i^{gt}$ is the ground truth density map.

### Evaluation metric

All the models are compared using the mean absolute error (MAE) metric which is commonly used in crowd counting research. Though there are alternate metrics available e.g., Grid Average Mean Error (GAME), MAE is a more common and standard metric. Also, any performance gain achieved using MAE also holds true for GAME^2,4^.

MAE is the average of absolute count error in predicted and actual density maps and is calculated using 12:

12 $$\begin{aligned} MAE = \frac{1}{N} \sum _{1}^{N}{(e_n - \hat{g_n})} \end{aligned}$$

where, *N* is number of images, $g_n$ is actual count and $\hat{e_n}$ is estimated count in the $n^{th}$ image.

## Results and discussion

The proposed method is evaluated over the benchmark datasets by training the three baseline models using standard training (without KD), and using the proposed method (with KD). To inspect the learning performance, the activation maps at different layers from both teacher and student models were analyzed to compare the model’s capabilities. An example of activation maps from a single layer from both teacher and student models are presented in Fig. 5.

**Table 1:** Counting accuracy performance of the proposed method on four benchmark datasets, using mean absolute error (MAE) metric.

Student $\downarrow$	Labelled data $40\%$		Labelled data $60\%$		Labelled data $80\%$		Labelled data $100\%$	
	Standard	Ours	Standard	Ours	Standard	Ours	Standard	Ours
Mall								
MCNN	9.8	8 (+ 18.3%)	6.1	5.2 (+ 14.9%)	4.8	4.2 (+ 11.6%)	3.6	3.3 (+ 8.0%)
DroneNet	9.3	7.6 (+ 17.9%)	6.5	5.4 (+ 16.3%)	4.5	3.9 (+ 12.5%)	3.3	3 (+ 9.6%)
LCDnet	9.9	8.5 (+ 14.3%)	7.2	6.2 (+ 13.6%)	5.6	4.9 (+ 12.2%)	4.2	3.8 (+ 8.8%)
ShanghaiTech Part A								
MCNN	140.2	103.7 (+ 26.0%)	125.4	94.6 (+ 24.6%)	118.6	96.9 (+ 18.3%)	110.2	91.9 (+ 16.6%)
DroneNet	128.8	101.9 (+ 20.9%)	114.8	93.6 (+ 18.5%)	105.7	88.6 (+ 16.2%)	98.5	83.9 (+ 14.8%)
LCDnet	156.3	128.5 (+ 17.8%)	134.5	111.5 (+ 17.1%)	127.4	110.2 (+ 13.5%)	118.4	107.9 (+ 8.9%)
ShanghaiTech Part B								
MCNN	40.5	29.8 (− 26.4%)	33.3	25.9 (− 22.2%)	29.1	24.3 (− 16.5%)	26.4	22.3 (− 15.5%)
DroneNet	38.6	31.1 (− 19.5%)	32.5	27.2 (− 16.2%)	24.8	21.6 (13.1%)	22.4	19.6 (− 12.6%)
LCDnet	44.3	34.5 (− 22.1%)	41.8	33.2 (− 20.6%)	35.9	29.7 (− 17.3%)	31.6	26.3 (− 16.8%)
DroneRGBT								
MCNN	36.4	27.5 (+ 24.5%)	28.5	22.9 (+ 19.6%)	21.6	17.6 (+ 18.4%)	17.9	15.7 (+ 12.3%)
DroneNet	33.7	25 (+ 25.7%)	22.9	17.8 (+ 22.3%)	18.7	14.8 (+ 20.6%)	11.3	9.7 (+ 14.5%)
LCDnet	38.5	30.8 (+ 19.9%)	29.3	23.9 (+ 18.3%)	24.6	20.5 (+ 16.8%)	21.4	18.4 (+ 14.2%)

**Fig. 6:**
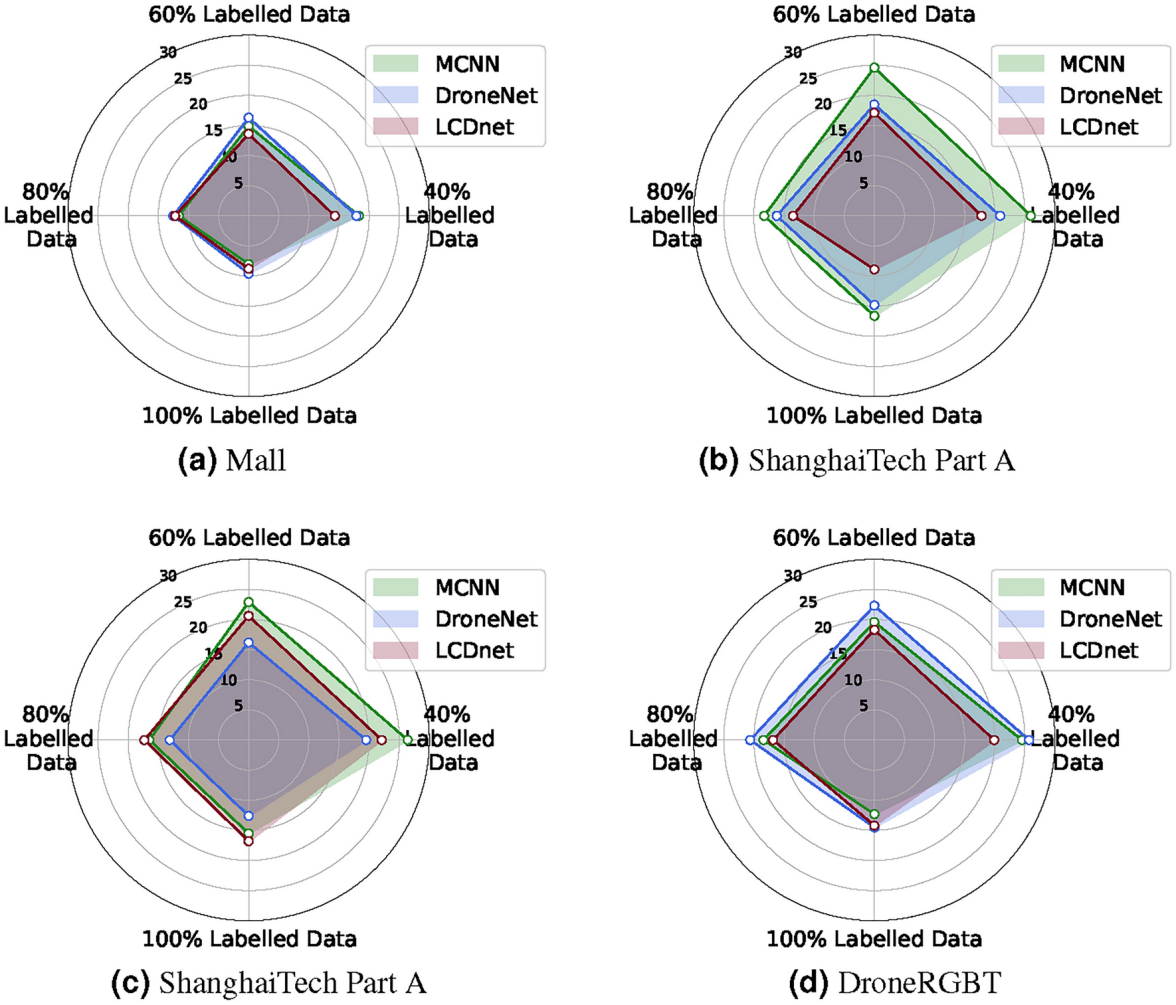
Percent (%) reduction in mean absolute error (MAE) using the proposed scheme against the respective baseline models for four different datasets.

Table 1 presents the counting performance of the proposed method over the four benchmark datasets. The mean absolute error (MAE) is compared against baseline models. Figure 6 illustrates the comparison more intuitively using spider charts. The figure depicts a percent reduction in MAE using the proposed scheme with respect to all baseline models. More specifically, each spider subplot represents the percent reduction in MAE of the three student models (MCNN, DroneNet, and LCDnet) when the proposed scheme is used. The reduction is reported for four different cases (Table 1). The analysis of the results shows that: (i) MCNN achieves higher gains on all datasets except on DroneRGBT in a few cases (at 40%, 100%). This is a model-specific observation that shows better learning or generalization capabilities of MCNN in most scenarios. However, DroneNet and LCDnet using KD achieve higher gains on the DroneRGBT dataset in a few cases. The results should not be confused with the overall accuracy (or error performance) of the models, but these only reflect the gains using the proposed scheme. For instance, DroneNet outperforms MCNN on almost all datasets. (ii) The performance gains for all models are typically maximum at the 40% labeled data and minimum at 100% labeled data. This is an expected and most significant result that was expected. This means that when the amount of labeled data is reduced, the baseline models start underperforming and the MAE increases. At the same time, by applying the proposed scheme, the student models learn from the teacher’s knowledge to improve their performance (MAE reduces). This results in reduced MAE using baseline models (using the proposed scheme) as compared to the baseline models (without KD), ultimately resulting in higher percent gains (Fig. 6). (iii) The percent gains are minimal for the Mall dataset as compared to the other dataset due to the lower MAE values (high accuracy) for the baseline models and thus less room for improvement to reflect in the percent gains.

**Fig. 7:**
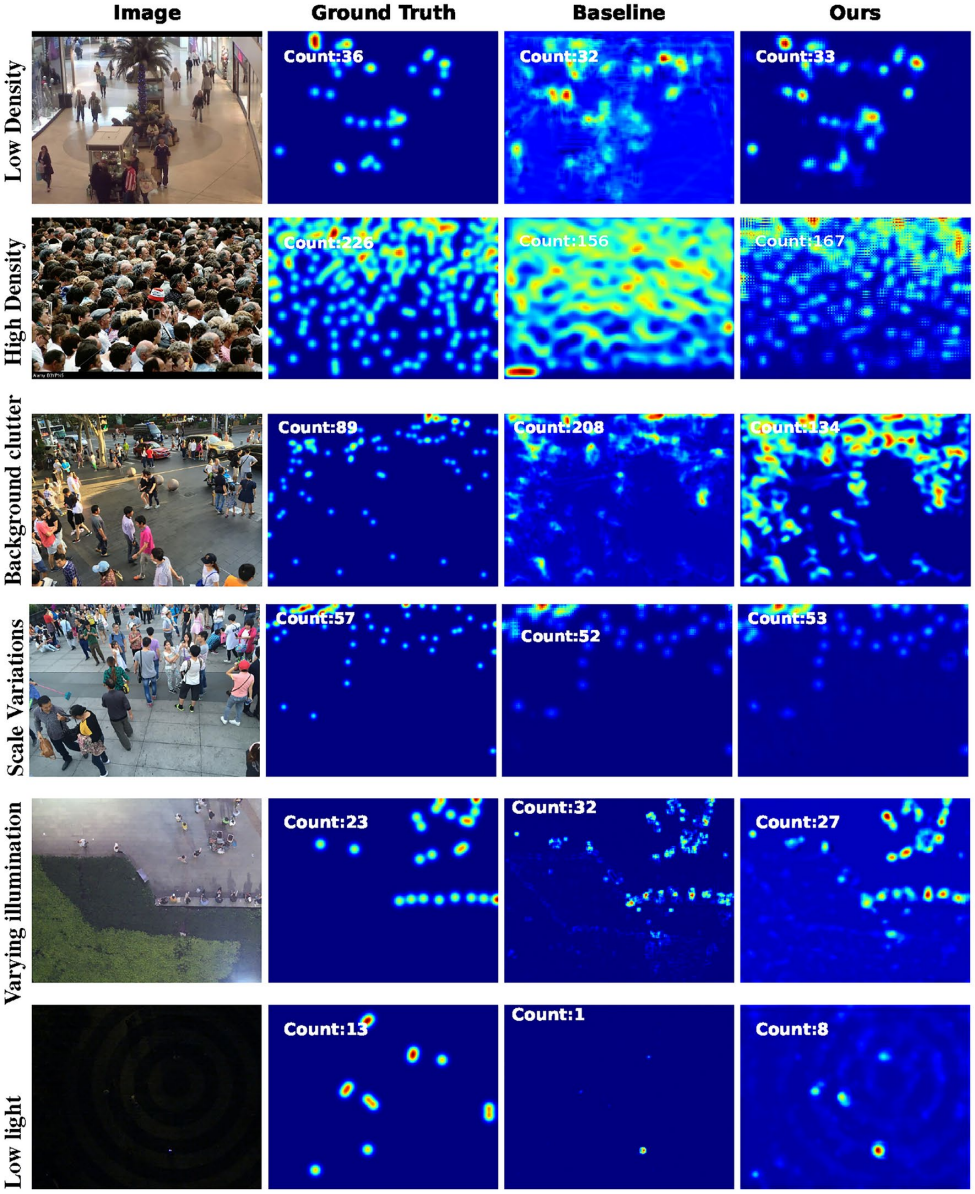
Predictions results on the sample images from Mall, ShanghaiTech Part A, Part B, and DroneRGBT datasets.

Furthermore, the LCDnet model is an extremely lightweight architecture with only 0.05M parameters as compared to MCNN (0.13M) and DroneNet (0.15). LCDnet also uses only two columns (3 columns in MCNN and DroneNet), with fewer layers. Thus, the relative lower accuracy (or higher MAE) of LCDnet is justified. Similarly, DroneNet achieves superior performance than MCNN due to the fact that DroneNet uses more powerful non-linear self-ONN operations rather than the linear convolution operations of CNN, in a similar architecture as MCNN. However, it is worth noting that the purpose of the study is not to evaluate the individual capabilities of different models to learn from KD but rather to evaluate the overall efficacy of KD in training lightweight crowd counting models of which MCNN, DroneNet, and LCDnet are examples. Figure 7 presents a single prediction output for a single sample from each dataset (Mall, ShanghaiTech Part A, Part B, and DroneRGBT).

## Computational complexity

Although the study aims to investigate knowledge distillation and its efficacy in training shallow student models since the final student models are designed to be deployed at the edge, it is significant to analyze the computational complexity of these models. Table 2 reports the computational complexity of the teacher and student models to understand why efficient shallow models are more suitable for edge devices, especially in real-time or near real-time applications. The analysis shows that the inference time of MCNN and DroneNet is reduced by a factor of 8 compared to the teacher model, while LCDnet achieves an 18-fold reduction. Similarly, the storage size of these student models is negligible (less than 1 MB) as compared to the teacher model (65 MB).

**Table 2:** Computational complexity of the student models trained with KD in terms of number of parameters (in Million), GMACs, size (in MB), and inference time (in mili seconds) for fixed input size.

Model	Output	Parameters(M)	Size(MB)	GMACs	Inference time(s)		
					Server	Jetson Xavier	Jetson Nano
Teacher model							
CSRNet^27^	1/8	16.26	65.05	135.4	0.19	1.01	1.88
Student models							
MCNN^26^	1/4	0.13	0.53	8.82	0.05	0.10	0.21
DroneNet^26^	1/4	0.15	0.60	8.92	0.056	0.15	0.28
LCDnet	1/2	0.05	0.21	4.85	0.006	0.05	0.10

## Ablation study

The experiments and results discussed earlier comprised people crowds (surveillance and aerial views). This section aims to validate the results achieved in the aforementioned section by further ablation studies. Specifically, we aim to evaluate the proposed method using different a teacher model, and datasets of nun-human objects (e.g., people, animals).

### Different teachers models

We use the CSRNet_lite^2^ model as a teacher to replace the CSRNet model (used in the earlier experiment) to validate the performance gains using different teacher models. The results are reported in Table 3.

**Table 3:** Ablation study using CSRNet_lite as a teacher with $60\%$ labelled data.

Models	Mall	ST Part A	ST Part B	DroneRGBT
MCNN	+ 1.3 $\uparrow$	− 3.6 $\uparrow$	+ 2.2 $\uparrow$	+ 1.3 $\uparrow$
DroneNet	+ 0.7 $\uparrow$	− 2.8 $\uparrow$	+ 1.2 $\uparrow$	+ 0.9 $\uparrow$
LCDnet	+ 0.8 $\uparrow$	− 1.2 $\uparrow$	− 0.7 $\uparrow$	+ 1.1 $\uparrow$

The $\downarrow \uparrow$ signs indicate the decrease/increase with respect to the baseline model, whereas the ± sign indicates an increase/decrease in MAE as compared to CSRNet as a teacher.

The results show that the proposed scheme outperforms baseline models even for the new teacher model (i.e., CSRNet_lite). Furthermore, CSRNet_lite also achieves slightly better accuracy (lower MAE) than CSRNet. These results signify the importance of the selection of a better teacher model to improve the performance of the student model.

### Cross-domain adaptation

Previously, we evaluated our proposed models on human crowds. We further studied the cross-domain adaptation using KD using two datasets of cars^66^ and sheep crowds^68^. The results are reported in Tables 4 and 5, respectively.

**Table 4:** Ablation study for domain adaptation on Sheeps dataset.

Models	MAE		SSIM		PSNR	
	Baseline	Ours	Baseline	Ours	Baseline	Ours
MCNN	5.76	4.13	0.88	0.92	28.6	30.94
DroneNet	5.11	4.06	0.90	0.94	29.9	32.15
LCDnet	7.82	5.36	0.76	0.85	25.47	29.02

**Table 5:** Ablation study for domain adaptation on CARPK dataset.

Models	MAE		SSIM		PSNR	
	Baseline	Ours	Baseline	Ours	Baseline	Ours
MCNN	10.20	8.05	0.76	0.80	19.6	24.22
DroneNet	9.10	7.67	0.81	0.85	21.08	25.83
LCDnet	13.05	10.18	0.71	0.79	20.8	23.44

**Fig. 8:**
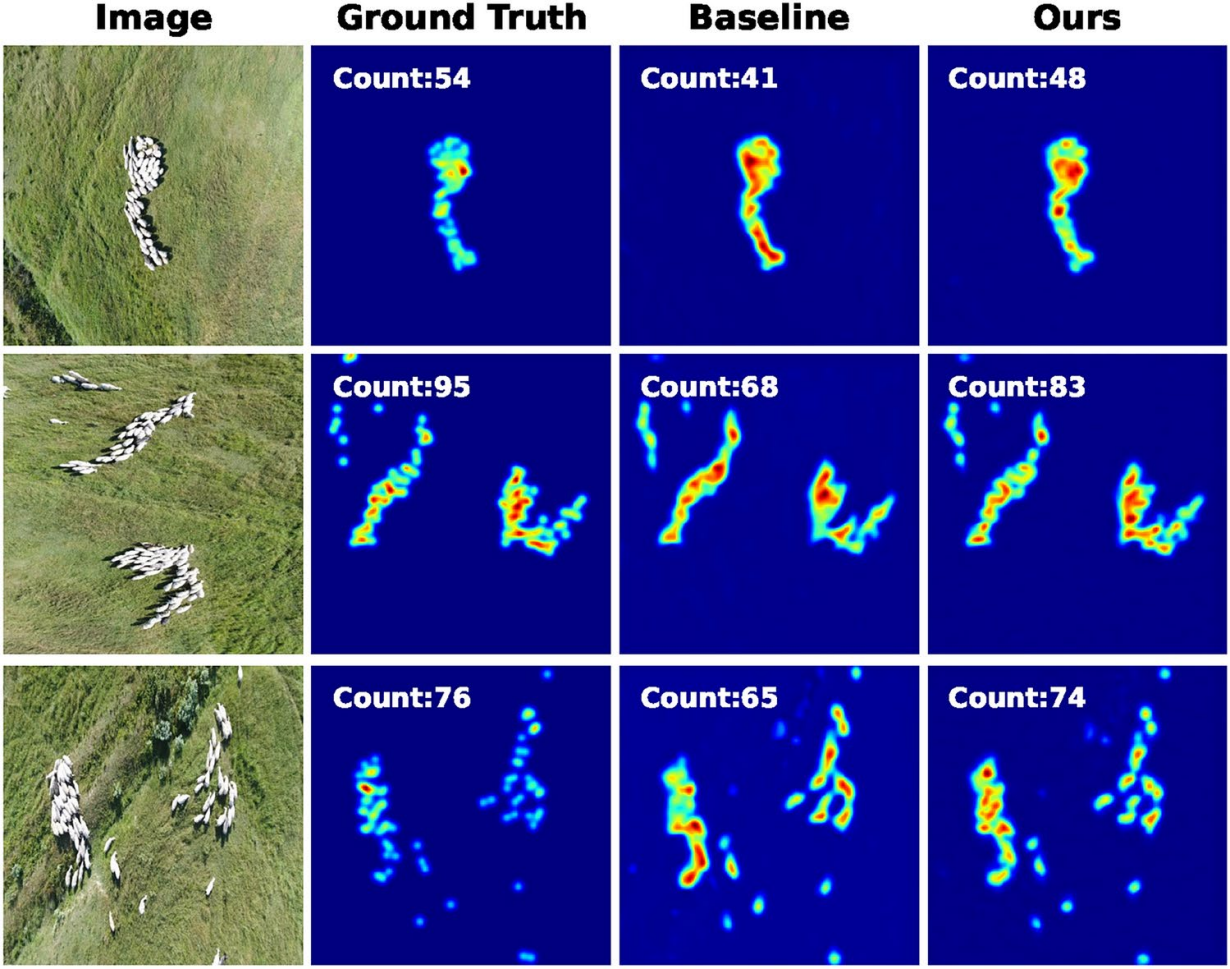
Predictions results on the Sheeps dataset.

**Fig. 9:**
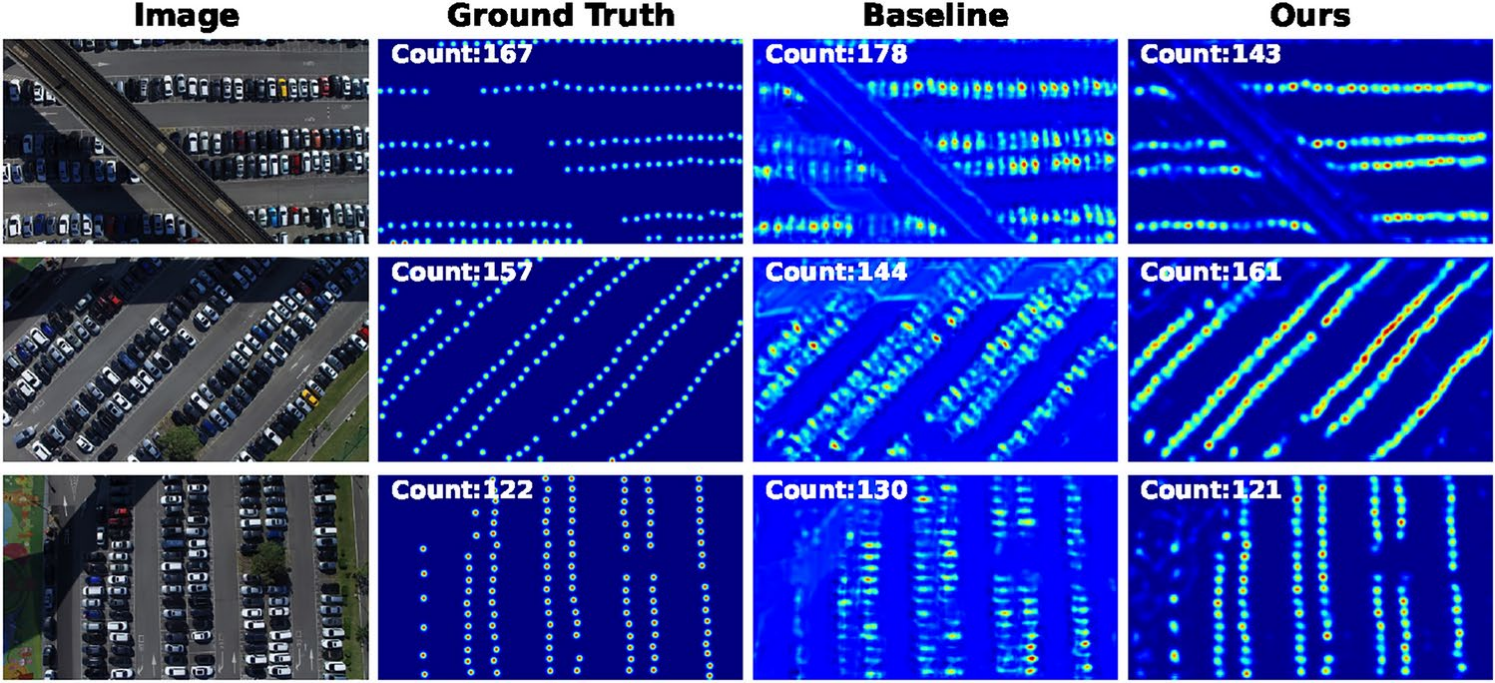
Predictions results on the CARPK dataset.

The results reported in Tables 4 and 5 further validate the results reported in section “Results and discussion” and dictate that the KD framework is both data-agnostic and model-agnostic. Furthermore, we introduced two new metrics i.e., structural similarity index (SSIM) and pixel signal-to-noise ratio (PSNR) as in^2,27,70^ to measure the quality of predicted density maps alongside the MAE metric for counting accuracy (or error) performance. The results show that the proposed method outperforms over the two datasets using all metrics.

Figures 8 and 9 depict sample predictions on the Sheep^68^ and CARPK^66^ datasets, respectively.

## Conclusion

This paper proposed the use of knowledge distillation (KD) to train compact crowd-counting architectures. KD improves the performance and accuracy of the compact model by distilling the dark knowledge from a cumbersome model during the training stage. It enhances the learning capability of the compact model by guiding the network to learn more fine-grained features under the supervision of the cumbersome model. KD has been applied in several computer vision and other deep learning tasks, but very few works exist on the use of KD in regression tasks such as crowd density estimation. This paper provides an intuitive framework with rigorous experimental analysis alongside ablation studies on domain adaptation and different teacher and student models to validate the performance gains. In future work, we aim to extend this study to integrate KD in federated learning (FL) to develop two-way privacy-preserving learning between student models (local models in the edge devices) and the teacher model (global model on the server). We also aim to investigate a novel case of server-less FL architecture with KD across edge devices.

## Data Availability

The Mall dataset analyzed during the current study is publicly available at: https://personal.ie.cuhk.edu.hk/~ccloy/downloads_mall_dataset.html The ShanghaiTech dataset analyzed during the current study is publicly available in the Github repository, https://github.com/desenzhou/ShanghaiTechDataset. The CARPK dataset analyzed during the current study is publicly available at https://lafi.github.io/LPN/. The DroneRGBT dataset analyzed during the current study is publicly available in the Github repository: https://github.com/VisDrone/DroneRGBT The Aerial Sheeps dataset analyzed during the current study is publicly available in the Github repository: https://huggingface.co/datasets/keremberke/aerial-sheep-object-detection.
